
JOURNAL INFORMATION
==============================
NLM Title Abbreviation: ScientificWorldJournal
Journal ID: ScientificWorldJournal
NLM Journal ID: 101131163
Journal ID: TSWJ

The Scientific World Journal

ISSN: 2356-6140
EISSN: 1537-744X
Publisher: Wiley

ARTICLE INFORMATION
==============================
PMCID: PMC5956373
PMID: 15349552
DOI: 10.1100/tsw.2004.79
Article ID: 728274
Article version: 1
Subjects: Research Article

Percutaneous Management of Urinary Calculi

Ramakumar Sanjay
Segura Joseph W.
Mayo Clinic, Rochester, Minnesota, USA
Academic Editor: Anthony Atala

Publication date: 2004
Electronic publication date: 2004 Jun 7
Volume: 4
First page: 296
Last page: 307
Copyright: Copyright © 2004 Sanjay Ramakumar and Joseph W. Segura.
Copyright year: 2004
License: This is an open access article distributed under the Creative Commons Attribution License, which permits unrestricted use, distribution, and reproduction in any medium, provided the original work is properly cited.
License URL: https://creativecommons.org/licenses/by/3.0/

==============================
Article
*TheScientificWorldJOURNAL* (2004) 4 (S1), 296–307
ISSN 1537-744X; DOI 10.1100/tsw.2004.79

### Percutaneous Management of Urinary Calculi

Sanjay Ramakumar, M.D. and Joseph W. Segura, M.D.
*Mayo Clinic, Rochester, Minnesota*

**E-mails**: sramakum@email.arizona.edu

*Previously published in the Digital Urology Journal*

**DOMAIN**: urology

### INTRODUCTION

The revolution of minimally invasive surgery began in 1976 when Fernström and Johannson performed the first percutaneous nephrolithotomy (PNL).[1] Urologists then began to realize the potential of renal surgery through small percutaneous tracts, and have refined the procedure into what is routinely practiced throughout the world today. Progress in this area has ultimately benefited patients, with reduced morbidity, convalescence and recovery. In the United States, endourology was pioneered by Smith and associates, who, in 1979, removed renal and ureteral stones through the percutaneous route.[2]

Recent advances in fiberoptic technology have allowed easier and safer access to the upper urinary tract from a retrograde approach. Another innovation in stone management is shockwave lithotripsy, which has been shown to be both safe and effective. However, there is a definite role for percutaneous management of urinary calculi in today's world.[3-9] Developments in stone fragmentation, newer instruments, and improved fluoroscopy have increased the versatility of percutaneous surgery. Flexible, steerable nephroscopes have allowed access to all parts of the kidney. The indications for open surgery in stone disease have thus become rare. In 1990, only 1-2% of urinary stones were treated by open surgery.[5] Today, this number is even smaller. This article will discuss the selection of the proper patient, as well as techniques of stone removal, and will review the advances and outcomes of percutaneous endourologic surgery in the last twenty years.

### PATIENT SELECTION AND PREPARATION

The key to a successful urologic intervention is not only the skill of the operator, but using proper judgment in selecting patients. Today's indications for percutaneous nephrolithotomy have arisen from the contraindications to ESWL as well as recognition of those situations where ESWL is less successful.[10,11] These include body habitus not allowing ESWL, cystine stones, stones associated with distal obstruction or foreign bodies, large or complex stones or ESWL failures.[12] Perhaps the only true contraindications to PNL is an irreversible bleeding diatheses. Nephrolithotomy should also be performed only when the urinary tract is free from infection. In cases of struvite calculi, this may not be possible and appropriate antibiotic coverage is necessary.

Radiologic studies necessary for PNL are a plain x-ray (KUB) and usually an intravenous pyelogram delineating renal anatomy. In special circumstances, computerized tomography is required to determine if a safe window is present to form the percutaneous tract. Evaluation of the stone itself can be divided into three categories: stone burden, stone location and stone composition.[13] Retrospective analyses of ESWL patients have demonstrated a higher incidence of failure and need for additional procedures when the stone burden is greater than 3 cm in diameter.[14]

Treatment of lower pole nephrolithaisis remains controversial.[15] Lingeman, et. al published a retrospective review and meta-analysis of the literature demonstrating a 90% stone free rate with PNL compared to 59% with ESWL. In contrast to this, a more recent study from Cass[16] recommends ESWL for lower pole stones smaller than two centimeters. His analysis reveals a lower stone-free rate than PNL, offset by a better retreatment and complication rate. An interesting technique of calyceal irrigation during ESWL for lower pole calculi has been described, but this is still investigational.[17] Further studies indicate that cystine stones are relatively resistant to shockwave therapy.[18]

Generally, the only preparation required is intravenous antibiotics, usually a cephalosporin. Especially in cases of struvite stones, broader coverage is utilized with ampicillin and gentamicin. No bowel preparation is necessary, and in cases of perforation (discussed later) conservative management is effective. An analysis of coagulation factors is also warranted.

### PERCUTANEOUS ACCESS

The endourologist is only as good as the access tract allows. Key placement of percutaneous tract or tracts can make the difference between safe, efficient stone removal and frustrating failure.[19] Who performs the access procedure is dependent on personal preference. At our institution, the access is obtained in the radiological suite, after which the patient is transferred to the operating room. For the access procedure, patients are placed in the prone position with mild intravenous sedation.[20] The previously obtained radiologic studies should delineate the relationship between the stone and the collecting system. If the stone is located in a particular calyx or diverticulum, access should be through that particular calyx or diverticulum.[21] If this is not the case, a lateral or posterior calyx is optimal for stone extraction. In the United States, fluoroscopic guidance is typically used for access, but European interventionists prefer ultrasound guidance.

Once the collecting system is entered, contrast is used for proper placement of the guide wires and later the nephrostomy tube, which at our institution is usually a Cope catheter secured to the skin. Retrograde assisted renal access has been described.[22,23] Using a flexible ureteroscope, the calyx is punctured and the wire is brought out through the skin. Snare wires have also been used for this technique.[24] Wires can be replaced and the tract dilated in the usual fashion. This is more difficult and not recommended in patients with staghorn or large volume stones.

Upper calyceal stone present a different twist for access. An intercostal approach is sometimes necessary, but pulmonary complications are significantly increased.[25] Karlin and Smith have described a technique to displace the kidney caudally using an Amplatz sheath in a lower or central calyx.[26] This brings the upper calyces below the twelfth rib and thus decreases the risk of pleural or pulmonary injury. Spinal and intrapleural bupivacaine has been used for postoperative analgesia for patients needing an intercostal access tract.[27]

In a morbidly obese patient, access may be achieved in the flank position.[28] An occlusive balloon catheter is placed in a retrograde fashion prior to antegrade percutaneous nephrostomy. This position allows for less restriction of chest movements and pulls the abdominal pannus forward.

Patients who have had multiple renal procedures prior to PNL may present difficult access scenarios. A Collings knife has been reported as being helpful in establishing a tract that is difficult to dilate.[29] However, significant bleeding may be encountered with this technique. Finally, transilial access was performed in a patient with crossed fused renal ectopia.[30] An existing ileal bone window created for posterior iliopsoas transplantation was utilized to gain access to the fused kidney without complication.

### TECHNIQUE OF STONE REMOVAL

We prefer our patients to have general anesthesia for PNL and placed prone to allow ease in positioning. Our technique has been previously described[5,31,32] and is briefly summarized as follows.

After the guide wire is placed down the ureter in the radiology suite, a straight, angiographic catheter is placed over the wire and the patient is transported to the operating room. The catheter is removed and the tract is dilated over the guide wire. The dilator used is a matter of personal preference. We prefer rapid dilation to 24-28F using flexible fascial dilators because they seem to have a better sense of feel. However, there is a higher chance of buckling in the retroperitoneum, especially when there is significant perinephric scarring or marked obesity. At that point we would use the telescoping metal dilators. The dilation procedure must be done under fluoroscopic guidance to assure proper placement into the renal pelvis. It is mandatory to have a safety guide wire in the ureter and perform the dilations over a second "working" wire. The nephroscope may be placed directly into the kidney through the tract or alternatively, an Amplatz sheath may be used.

Smaller stones may be removed using forceps or a basket, but in general the larger stones must be fragmented prior to extraction. We prefer the ultrasonic lithotrite because of its power to fragment and its simultaneous ability to suction stone particles. Alternative methods of fragmentation are the electrohydraulic probe, which works especially well for harder stones (cystine and calcium oxalate monohydrate), the holmium:YAG laser[33] and the Swiss lithoclast.[34,35]

Flexible nephroscopy may be employed in cases of stone fragments migrating to other calyces or additional stone burden. The laser fiber is very useful in this situation. When proficiency is reached with PNL techniques, bilateral procedures may done with only minimal increases in morbidity.[36]

The recent summary report from the ureteral stones guidelines panel[37] has the following recommendations: Stones in the upper ureter less than one centimeter should have ESWL as a first line of therapy. Ureteroscopy and PNL are acceptable choices in situations where ESWL is not feasible or has failed. Stones greater than one centimeter can be treated with either ESWL, ureteroscopy or PNL. In an age of ureteroscopy, lower ureteral stones are no longer in the realm of PNL.

At the conclusion of a PNL procedure, a basket is often placed down the ureter under fluoroscopic guidance to retrieve any fragments that may have traveled down. A 6F angiographic catheter is placed into the ureter and a 22F Foley is used as a nephrostomy tube with 1-2 cc in the balloon. A nephrostogram is performed in 24-48 hours and the tubes are removed if there is no extravasation or retained calculi.

Staghorn calculi often present increased difficulty.[38,39] PNL is particularly useful in the management of these stones which are usually made of struvite. The internal anatomy of the kidney determines whether one or several tracts will be necessary. Too many dendritic extensions or obstructed calyces may mean that PNL alone is not be the best option in the particular stone. The collecting system must be inspected at the end of the procedure using a flexible nephroscope to locate any residual stony material. Many struvite staghorn calculi are treated very well by "sandwich therapy"[40] or combination therapy, which involves a PNL session followed by ESWL, and a subsequent PNL session to remove any remaining debris. The stone free rates are comparable to open surgery and PNL alone. A set of guidelines was created by the Nephrolithaisis Clinical Guidelines Panel for the management of staghorn calculi.[41] The committee believes that a newly diagnosed staghorn is an indication for treatment. As a guideline, PNL followed by ESWL or repeat PNL should be used for most standard patients with struvite staghorns. As a guideline, neither SWL monotherapy nor open surgery should by used as first-line treatment for staghorns in most standard patients. As options, PNL and ESWL are equally effective in treating small-volume staghorns when the renal anatomy is normal or near normal. Also as an option, open surgery is appropriate therapy when the staghorn cannot be managed by any reasonable number of PNL sessions or ESWL sessions. Nephrectomy is a reasonable option for a poorly functioning stone-bearing kidney.

Cystine calculi are generally resistant to shockwave therapy and thus, medical failures are best suited for PNL. The long hospitalizations associated with dissolution therapy preclude its use as primary therapy.[42]

### RESULTS

The literature demonstrates that over the years, proficiency with PNL has improved dramatically with experience. Our first 15 cases were preliminarily reported in 1982.[43] All stones were under 2 cm and removed successfully with an average hospital stay of 4 days. Another report of the efficacy of PNL came from Europe the following year.[44] An adequate tract was created in only 84% of cases and the stone free rate was 71%. The average hospital stay was 8.3 days. An important study from the United States from White and Smith in 1984 showed a 95% stone-free rate with renal stones and 80% success for ureteral calculi.[45] Hospital stay was 3 days and the complication rate was approximately 8.5%. One year later our institution presented a review of 1,000 PNL cases, citing successful removal of renal and ureteral stones in 98.3% and 88.2% respectively. The mean length of stay was 5.2 days (day of procedure = day 1) with a 3.2% complication rate. From that time, further reports followed showing results similar to open extraction with significantly decreased morbidity and convalescence.[46-51]

Long term stone-free status can be achieved using PNL.[52] When following patients on a long term basis, it has been shown that tomography is superior to plain radiography for the detection of renal stones.[53] Computerized tomography is more sensitive and specific than all other imaging modalities[54], however the cost effectiveness of this test precludes its routine use.

The invention of ESWL has dramatically changed the management of renal calculi. Early on, we evaluated the role of PNL and its indications, which have become a part of the indications urologists follow today.[14] Case controlled studies have documented the superior effectiveness of PNL compared to ESWL monotherapy.[55,56] Sandwich therapy has dramatically improved the clearance of renal stones and has reduced ancillary procedures for staghorn calculi. An important distinction for stone size was published by Lam and colleagues, comparing success after PNL alone, combination therapy, and ESWL monotherapy.[57] The initial stone-free rate for PNL alone was 84.2% compared to 51.2% for ESWL alone. For staghorn calculi smaller than 500 $mm^2$ the stone-free status improved to 94.4% for PNL with or without ESWL, compared to 63.2% for ESWL monotherapy. When the stone surface area increased to 1000 $mm^2$ the ESWL clearance rate dropped to 22.2%. Thus, shockwave therapy alone should be reserved for smaller staghorn calculi. Additional studies correlate with these findings.[58-62]

Struvite staghorn calculi are prone to recurrence, especially when residual fragments are present.[63] When an aggressive combined endourological approach is taken in the management of struvite calculi, long-term results are comparable to the standard open technique.[64] The only significant risk factor for stone recurrence is the presence of an associated anatomic anomaly.[65]

Medical management of cystine calculi is very effective, though obstructing or symptomatic calculi occasionally still require surgery. PNL has proven to the most effective intervention, superior to ESWL.[66] Cystine stones can also be subdivided between rough and smooth, the latter thought to be more resistant to shockwave therapy.[67] Initially it was felt that in the treatment of cystine staghorn stones, debulking, and medical management should resolve most cases. This has not proven to be true. Medical management is ineffective in removing residual stones. For optimal results, these patients should be made stone free at the time of PNL.[68]

### COMPLICATIONS

In the United States, fluoroscopy is used during tract formation and PNL. It is important that the radiation dose received by the surgeon is within the acceptable range.[69,70] Use of lead glasses, thyroid shield, as well as a lead apron are recommended if an overhead x-ray source is used. This is less important if an under the table C-arm is employed. Additional measures taken to reduce radiation exposure are limiting fluoroscopy time and coning down the field when possible.

The renal effects of PNL have been well documented in the literature.[71-73] There is certainly some scarring at the nephrostomy site, but $^{99m}$DTPA and $^{99m}$DMSA scintigraphy have proven that no loss of functioning renal parenchyma occurs with these scars. It is also important to note that multiple nephrostomy tracts did not cause increased renal damage. Enzymatic evaluation of kidneys using N-acetyl-glycosaminidase, a sensitive marker of renal tubular damage, does not show any significant change after PNL and ESWL.[74] Comparison of renal scars produced from ESWL and PNL by single photon emission computed tomography (SPECT) reveal that renal lesions seem to be greater from ESWL than from PNL, and patients requiring multiple shockwave treatments may be better served by PNL.[75] Finally, Wilson and co-workers performed an experimental comparison in pigs of the bioeffects of pyelotomy, nephrotomy, piezoelectric lithotripsy, and placement of a percutaneous nephrostomy tube with balloon dilation.[76] Percutaneous nephrostomy had statistically higher renal scarring than the other groups, but neither creatinine, PAH clearances, nor renal plasma flows changed significantly. Moreover, no change in renal function was observed after treatment when compared to baseline function.

Significant blood loss may occur when the nephrostomy tract is created, during PNL, and for some time afterwards. A retrospective analysis from Stoller, et. al. estimated the average blood loss from a one-stage single puncture PNL to be 2.8 gm/dL hemoglobin.[77] Multiple punctures and renal pelvic perforation doubled the blood loss. Patients with preexisting nephrostomy tracts had half of the blood loss seen with PNL. Factors such as puncture site, type of fascial dilator, hypertension, renal insufficiency, infection, previous open renal surgery or prior ESWL did not effect the estimated blood loss. Data from our institution in a review of 1000 patients shows an average blood loss of 1.2 g of hemoglobin with a transfusion rate of 3%.[78]

Bleeding during PNL may be enough to terminate the procedure. The technique we have used involves placement of a large nephrostomy tube, clamping it for 30-40 minutes and administering intravenous diuretics. This will stop most bleeding that is venous in origin. Occasionally arterial bleeding is encountered and has needed further therapy. Patterson and colleagues reviewed 1,032 PNL procedures and reported the incidence of significant, documented or presumed vascular injury to be 0.9%.[79] Pseudoaneurysm, arteriovenous fistula, or vascular laceration were all diagnosed angiographically and treated with transcatheter techniques successfully. Delayed bleeding was also seen and treated with conservative therapy. The recommendation for patients with serious postoperative bleeding is arteriography and embolization of peripheral vessels as opposed to open exploration. A hemostatic tamponade catheter has also been used with success.[80,81]

Extravasation of irrigating fluid can be a life threatening complication when it is unrecognized.[82,83] The operating room personnel must monitor the input of fluid versus the output from bladder drainage and loss at the table. When a difference of 500cc is reached, consideration should be given to terminating the procedure. Aggressive diuresis and careful electrolyte management are required. Absorption of cold irrigation fluid has more of an effect on thermoregulation than warmed solution.[84] Most patients will recover without long term sequela.

Any chest or abdominal organ has a chance of injury during nephrostomy tract creation and PNL. A supracostal approach for upper renal stones has a significantly increased rate of pulmonary complications.[85] Hydrothorax and pneumothorax are known complications, and patients must be informed of the possible need for a chest tube and/or further therapy. Most small pneumothoraces may be managed conservatively, provided that the patient is not symptomatic. Pleural extravasation of infected urine poses a greater threat to the patient.

Bowel injury is rare and thus patients are not routinely given a preoperative bowel preparation. Anatomic variations and prior bowel surgery most commonly contribute to bowel injury. Extraperitoneal colon injury can be managed conservatively by stenting the urinary system, and using the percutaneous tube as a colostomy tube.[86] When the connection between the urinary and fecal tracts has sealed, the tube can be removed. The cutaneous fistula tract will close shortly thereafter. Intraperitoneal perforation of the colon mandates open exploration and repair.[87] Duodenal injury has also been reported with PNL and was successfully managed conservatively.[88]

Left renal PNL has the risk of splenic injury, especially if splenomegaly is present.[89] It is rare, but a patient with known splenomegaly will be better served with an abdominal CT scan to identify a safe "window" for nephrostomy creation. Injury to the spleen usually requires exploration and splenectomy.

Patients with renal pelvic stones that are lodged at the UPJ have a higher incidence of UPJ obstruction following PNL.[90] It is sometimes difficult to ascertain whether the obstruction predated the PNL procedure.

Spinal cord injury patients are prone to autonomic dysreflexia, usually when the lesion is above T6. Stimuli such as bladder distension and constipation can trigger the sympathetic outflow with reflex bradycardia. This has also been reported during a PNL procedure.[91] The symptoms resolved when the procedure was terminated and the patient underwent an open pyelolithotomy without complication.

Removal of the nephrostomy tube should be performed with a clear understanding of its mechanics. Many times, fluoroscopic guidance is necessary to assure complete removal of locking catheters. The catheter should be thoroughly inspected to assure complete removal. Retained fragments predispose the patient to infection and calculi formation.[92]

Computerized tomography (CT) is an excellent way for delineating renal and perirenal morphology. Late sequela of PNL are rare and renal function remains constant.[93] Ultrasound guidance can be used for the detection of complications[94], but CT scanning remains the most sensitive test today. Overall, percutaneous nephrolithotomy is a safe and effective method of removing renal calculi. The complications are rare and most of them can be managed conservatively.[95-97]

### SPECIAL CIRCUMSTANCES

Calyceal diverticula are lined with non-secretory transitional epithelium and are connected to the normal collecting system by a narrow isthmus. They fill by the retrograde passage of urine from the calyx, though drainage may be impaired if the neck of the diverticulum is narrow. Often times the connection can be obliterated. They are thought to be incidental in less than 1% of people, congenital in origin and bilateral in 3%.[98] Though most calyceal diverticula are asymptomatic, they can present with flank pain, hematuria or recurrent infection, and thus require treatment.[99] Stones can form in them 10-50% of the time. A series of 10 patients who had diverticular stones were managed using ESWL by Psihramis and Dretler.[100] Although 8 of the 10 patients were not stone free, 70% of the group were asymptomatic after ESWL. It should be noted that the largest stone was 14 mm in their group. This form of treatment, however, does not eradicate the underlying pathology. Percutaneous management of these stones has been extremely successful.[101] The tract must enter the diverticulum directly and the neck should be dilated to prevent further stone formation if there is a substantial amount of overlying parenchyma.[99] The collecting system can be punctured from an antegrade approach if an opening is not seen. After any stones have been removed, the cavity should be fulgurated. Alternatively, the infundibulum may be dilated and a nephrostomy tube placed for a few days. When the parenchyma is thin, the diverticular wall as well as the isthmus should be fulgurated to obliterate the cavity and the roof of the diverticulum should be resected.[102] Difficulties will be encountered with anterior and superior calyceal diverticula. A newer technique has been described using a retrograde assisted percutaneous approach for improved operative time.[103] Lower calyceal diverticula may be more difficult to reach with the flexible ureteroscope.

Urolithaisis in a transplanted kidney is a rare occurrence. The reconstructed anatomy of the transplanted ureter reduces the likelihood of fragments passing after ESWL.[104] Percutaneous extraction of stones is a very safe and effective method of stone removal.[105-108] The anterior nature of the transplant offers easier access through the anterior abdominal wall and tract dilation. The collecting system can be localized easily with ultrasound guidance, especially when hydronephrosis is present. Other urologic complications of transplantation may be handled by this route also.

Patients with solitary kidneys are able to have PNL without any increase risk.[109] Renal function is not adversely affected and PNL may be performed even with renal insufficiency. Caution must be given to monitor the irrigation fluid balance at all times, as these patients may not be able to handle the fluid shifts.

PNL for calculi in horseshoe kidneys has been performed with success.[110] In fact, ESWL may be more difficult for two reasons. First, the anteromedial and inferior position of the horseshoe kidney makes it more difficult to focus the shockwave therapy. Second, stones in the medial calyces may be obscured by the spine, and the pelvic bones may obscure the lower calyceal stones. Modifications must be made to the PNL technique because of these anatomic changes.[111] The lower abdominal position of a horseshoe kidney necessitates upper or middle calyceal puncture, while the malrotation requires a more posterior puncture. Monitoring of the tract formation is difficult using fluoroscopy. Ultrasound guidance may be more helpful in this regard, and is also useful in locating aberrant vessels. The renal pelvis is deep and a long nephroscope may be required. Similarly, with a pelvic kidney, PNL may be possible using laparoscopy to displace the bowels.[112] A case has also been reported using "open" PNL for failed anatrophic nephrolithotomy.[113]

Morbid obesity that precludes placement in the ESWL tub or results in ESWL failure are indications for PNL. Carson and colleagues have shown that PNL can be performed in obese patients with success.[114] The technique in these patients, however, may need some modification.[115] It is important to measure the skin to stone distance to see if longer instruments may be needed. This can be done with CT scanning or ultrasound. To assist with PNL, two techniques are described. First, to gain additional length, the skin and subcutaneous fat are incised down to the fascia. The Amplatz sheath should be secured to the skin with a heavy suture to prevent loss into voluminous subcutaneous fat.[4] The second technique is a two-staged procedure. When the tract has matured for a week or two, a flexible cystoscope can be used. However, visibility is diminished with this scope. Giblin, et. al have suggested using a longer Amplatz sheath and a gynecologic laparoscope to reach the stones in a one-staged procedure.[115]

PNL may be performed in the elderly patient without increased complications. Stoller and colleagues reviewed their patients over 65 and compared them with younger patients.[116] No significant difference was seen as far as success or complications between the groups. The blood transfusion rate was higher in the elderly group despite similar preoperative hemoglobins. They hypothesize that the older patients have a lower threshold for transfusion given the increased risk for cardiac disease and thus an increased transfusion rate. Similarly, Horgan, et. al. have shown that endopyelotomy is safe in patients over 65.[117] They also had an increased transfusion rate (11%) and suggest that elderly patients pose a greater risk for blood loss during percutaneous procedures due to the effects of atherosclerosis on renal blood vessels. Patient positioning must be undertaken with care. Osteoporosis and debilitating arthritis may predispose to significant postoperative problems. Pain and post-procedure mobilization may be a problem. Patient education is very valuable to assist the elderly patient.

Turning to the other side of the age spectrum, PNL is possible in children and infants. A review of 53 cases of patients 16 years old or less by Kurzrock, et. al shows that there has not been any evidence of decreased renal growth or scarring with minimally invasive techniques.[118] Performing PNL in smaller kidneys does not lead to increased complications, either. In fact, combination therapy of PNL, ESWL and percutaneous irrigation has been accomplished in a 9 month old baby.[119]

Occasionally, the renal anatomy is distorted by benign structures. A technique of cyst puncture can shift the kidney to a more accessible position for PNL.[120] The cyst cavities in those cases should be drained percutaneously until all the drainage has stopped. Finally, patients with multiple benign tumors such as angiomyolipomas present a clinical dilemma. Open nephrolithotomy has a high risk for nephrectomy. One case is reported in the literature of PNL directly through an angiomyolipoma in a patient with tuberous sclerosis.[121] Fortunately, significant bleeding was not encountered and the authors advise that the PNL be performed with a Kaye balloon nephrostomy tube placed postoperatively.

### COST COMPARISON

It is difficult to predict the costs involved in renal stone removal when prices and outcomes across the world are variable. Chandhoke has created a cost-effectiveness model for the treatment of staghorn calculi and this represents the best model we have today for comparison.[122] Overall, PNL and combined sandwich therapy were more cost-effective than shock wave lithotripsy monotherapy. When the stone surface area was less than 500 $mm^2$, combined sandwich therapy and shock wave lithotripsy monotherapy were equal. However, when the stone burden exceeded 500 $mm^2$, combined therapy clearly became more cost-effective. This also reinforces the point that ESWL is at its best when only one treatment is required and the risk of secondary procedures is minimized. Further cost-effectiveness studies can hopefully characterize the treatment of all renal stones.

### FUTURE

PNL will always have a role in the management of urinary calculi. With constant improvements in optics and stone fragmentation, PNL continues to become more effective with fewer complications. In an era of shockwave therapy, there is a definite niche for percutaneous procedures. These have been refined over the years, but general indications for PNL are as follows: contraindication or failure of ESWL, staghorn calculi, large or lower pole stone burden, cystine calculi, abnormal renal or patient anatomy and the transplanted kidney.[104]

### REFERENCE LIST

1. Fernström, I. and Johannson, B.: Percutaneous pyelithotomy. A new extraction technique. Scand J Urol Nephrol, **10** : 257, 1976.
2. Smith, A. D., Reinke, D. B., Miller, R. P. and Lange, P. H.: Percutaneous nephrostomy in the management of ureteral and renal calculi. Radiology, **133**: 49, 1979.
3. Segura, J. W.: The role of percutaneous surgery in renal and ureteral stone removal. J. Urol., **141**: 780, 1989.
4. Segura, J. W.: Role of percutaneous procedures in the management of renal calculi. [Review] [24 refs]. Urol Clin North Am, **17**: 207, 1990.
5. Segura, J. W.: Current surgical approaches to nephrolithiasis. [Review] [20 refs]. Endocrinol Metab Clin North Am, **19**: 919, 1990.
6. Tan, E. C., Tung, K. H., Kwok, R. and Foo, K. T.: Percutaneous ultrasonic lithotripsy--its role in the management of renal and upper ureteric stones. Singapore Med J, **30**: 45, 1989.
7. Alken, P., Hutschenreiter, G., Gunther, R. and Marberger, M.: Percutaneous stone manipulation. J. Urol., **125**: 463, 1981.
8. LeRoy, A. J.: Diagnosis and treatment of nephrolithiasis: current perspectives [see comments]. [Review] [48 refs]. AJR Am J Roentgenol, **163**: 1309, 1994.
9. Ohlsen, H. and Kinn, A. C.: Percutaneous extraction of upper urinary calculi under fluoroscopic control--still a valuable complement to ESWL. Scand J Urol Nephrol, **27**: 311, 1993.
10. Carson, C. C. and Little, N.: The absolute and relative contraindications to ESWL. Problems in Urology, **1**: 580, 1987.
11. Motola, J. A. and Smith, A. D.: Therapeutic options for the management of upper tract calculi. [Review] [87 refs]. Urol Clin North Am, **17**: 191, 1990.
12. Streem, S. B.: Stone Extraction. In: Smith's Textbook of Endourology. Edited by Smith, A., Badlani, G., Bagley, D., Clayman, R., Jordan, G. H., Kavoussi, L. R., Lingeman, J. E., Preminger, G. M. and Segura, J. W. St. Louis: Quality Medical Publishing, Inc., vol. 1, Chapt. 18, pp. 239-263, 1997.
13. Van, A. N., Banner, M. P. and Pollack, H. M.: Radiographic imaging and urologic decision making in the management of renal and ureteral calculi. [Review] [41 refs]. Urol Clin North Am, **17**: 171, 1990.
14. LeRoy, A. J., Segura, J. W., Williams, H. J. and Patterson, D. E.: Percutaneous renal calculus removal in an extracorporeal shock wave lithotripsy practice. J. Urol., **138**: 703, 1987.
15. Lingeman, J. E., Siegel, Y. I., Steele, B., Nyhuis, A. W. and Woods, J. R.: Management of lower pole nephrolithiasis: a critical analysis. J. Urol., **151**: 663, 1994.
16. Cass, A. S.: Extracorporeal shockwave lithotripsy or percutaneous nephrolithotomy for lower pole nephrolithiasis? J Endourol, **10**: 17, 1996.
17. Graham, J. B. and Nelson, J. B.: Percutaneous caliceal irrigation during extracorporeal shock wave lithotripsy for lower pole renal calculi. J. Urol., **152**: 2227, 1994.
18. Zhong, P. and Preminger, G. M.: Mechanisms of differing stone fragility in extracorporeal shockwave lithotripsy. J Endourol, **8**: 263, 1994.
19. Lee, W. J.: Advances in percutaneous nephrostomy. [Review] [48 refs]. Yonsei Med J, **31**: 285, 1990.
20. LeRoy, A. J., May, G. R., Bender, C. E., Williams, H. J., McGough, P. F., Segura, J. W. and Patterson, D. E.: Percutaneous nephrostomy for stone removal. Radiology, **151**: 607, 1984.
21. Segura, J. W.: Percutaneous Management. In: Campbell's Urology. Edited by Walsh, P. C., Retik, A. B., Stamey, T. A. and Vaughan, E. J. Philadelphia: W. B. Saunders Company, vol. 3, Chapt. 60, pp. 2183-2194, 1992.
22. Grasso, M., Lang, G. and Taylor, F. C.: Flexible ureteroscopically assisted percutaneous renal access. Tech Urol, **1** : 39, 1995.

23. Spirnak, J. P. and Resnick, M. I.: Retrograde percutaneous nephrostomy. Urol Clin North Am, **15**: 393, 1988.
24. Nosher, J. L., Siegel, R. L. and Bodner, L. J.: Use of a snare wire to perform nephrostomy access in the presence of obstructive staghorn calculi. Cardiovasc Intervent Radiol, **19**: 193, 1996.
25. Narasimham, D. L., Jacobsson, B., Vijayan, P., Bhuyan, B. C., Nyman, U. and Holmquist, B.: Percutaneous nephrolithotomy through an intercostal approach. Acta Radiol, **32**: 162, 1991.
26. Karlin, G. S. and Smith, A. D.: Approaches to the superior calix: renal displacement technique and review of options [see comments]. J. Urol., **142**: 774, 1989.
27. Saied, M. M., Sonbul, Z. M., el-Kenawy, M. and Atallah, M. M.: Spinal and interpleural bupivacaine for percutaneous nephrolithotomy. Middle East J Anesthesiol, **11**: 259, 1991.
28. Kerbl, K., Clayman, R. V., Chandhoke, P. S., Urban, D. A., De, L. C. and Carbone, J. M.: Percutaneous stone removal with the patient in a flank position. J. Urol., **151**: 686, 1994.
29. Davis, B. E., Noble, M. J. and Mebust, W. K.: Use of the Collings knife electrode for percutaneous access in difficult endourology cases. J. Urol., **145**: 257, 1991.
30. Siegel, Y. I. and Lingeman, J. E.: Percutaneous transilial access for stone removal in crossed fused renal ectopia. Urology, **42**: 82, 1993.
31. Segura, J. W., Patterson, D. E., LeRoy, A. J., May, G. R. and Smith, L. H.: Percutaneous lithotripsy. J. Urol., **130** : 1051, 1983.
32. LeRoy, A. J. and Segura, J. W.: Percutaneous removal of renal calculi. Radiologic Clinics of North America, **24**: 615, 1986.
33. Razvi, H. A., Denstedt, J. D., Chun, S. S. and Sales, J. L.: Intracorporeal lithotripsy with the holmium: YAG laser [see comments]. J. Urol., **156**: 912, 1996.
34. Denstedt, J. D.: Use of Swiss Lithoclast for percutaneous nephrolithotripsy. J Endourol, **7**: 477, 1993.
35. Denstedt, J. D.: Percutaneous renal stone removal using the Swiss Lithoclast. Rev Med Suisse Romande, **112**: 725, 1992.
36. Ahlawat, R., Banerjee, G. K. and Dalela, D.: Bilateral simultaneous percutaneous nephrolithotomy. A prospective feasibility study. Eur Urol, **28**: 116, 1995.
37. Segura, J. W., Preminger, G. M., Assimos, D. G., Dretler, S. P., Kahn, R. I., Lingeman, J. E. and Macaluso, J. J.: Ureteral Stones Clinical Guidelines Panel Summary: Report on the Management of Ureteral Calculi. J. Urol., **158**: 1915, 1997.
38. Segura, J. W.: Staghorn calculi. [Review] [28 refs]. Urol Clin North Am, **24**: 71, 1997.
39. Chibber, P. J.: Percutaneous nephrolithotomy for large and staghorn calculi. J Endourol, **7**: 293, 1993.
40. Streem, S. B.: Sandwich therapy. [Review] [33 refs]. Urol Clin North Am, **24**: 213, 1997.
41. Segura, J. W., Preminger, G. M., Assimos, D. G., Dretler, S. P., Kahn, R. I., Lingeman, J. E., Macaluso, J. J. and McCullough, D. L.: Nephrolithiasis Clinical Guidelines Panel summary report on the management of staghorn calculi. The American Urological Association Nephrolithiasis Clinical Guidelines Panel. J. Urol., **151**: 1648, 1994.
42. Aabech, J. and Andersen, J. T.: Treatment of cystine stones: combined approach using open pyelolithotomy, percutaneous pyelolithotripsy, extracorporeal shock wave lithotripsy and chemolysis. Scand J Urol Nephrol, **27**: 415, 1993.
43. Segura, J. W., Patterson, D. E., LeRoy, A. J., McGough, P. F. and Barrett, D. M.: Percutaneous removal of kidney stones. Preliminary report. Mayo Clinic Proceedings, **57**: 615, 1982.
44. Wickham, J. E., Kellett, M. J. and Miller, R. A.: Elective percutaneous nephrolithotomy in 50 patients: an analysis of the technique, results and complications. J. Urol., **129**: 904, 1983.
45. White, E. C. and Smith, A. D.: Percutaneous stone extraction from 200 patients. J. Urol., **132**: 437, 1984.
46. Reddy, P. K., Hulbert, J. C., Lange, P. H., Clayman, R. V., Marcuzzi, A., Lapointe, S., Miller, R. P., Hunter, D. W., Castaneda-Zuniga, W. R. and Amplatz, K.: Percutaneous removal of renal and ureteral calculi: experience with 400 cases. J. Urol., **134**: 662, 1985.
47. Brannen, G. E., Bush, W. H., Correa, R. J., Gibbons, R. P. and Elder, J. S.: Kidney stone removal: percutaneous versus surgical lithotomy. J. Urol., **133**: 6, 1985.
48. Henriksson, C., Geterud, K., Grenabo, L., Hedelin, H., Naucler, J., Pettersson, S. and Zachrisson, B. F.: Percutaneous renal and ureteric stone extraction. Report on the first 500 operations. Scand J Urol Nephrol, **23**: 291, 1989.
49. el-Kenawy, M. R., el-Kappany, H. A., el-Diasty, T. A. and Ghoneim, M. A.: Percutaneous nephrolithotripsy for renal stones in over 1000 patients. Brit. J. Urol., **69**: 470, 1992.
50. Karsza, A., Heggagi, M. A. and Magasi, P.: Evaluation of 1000 percutaneous nephrostomies with a special view to efficiency and complications. Acta Chir Hung, **32**: 355, 1991.
51. Li, M. K., Wong, M. Y., Toh, K. L., Ho, G. H. and Foo, K. T.: Percutaneous nephrolithotomy--results and clinical experience. Ann Acad Med Singapore, **25**: 683, 1996.
52. Henriksson, C., Geterud, K., Pettersson, S. and Zachrisson, B. F.: Stone recurrences in kidneys made stone-free by percutaneous extraction. Scand J Urol Nephrol, **27**: 151, 1993.
53. Denstedt, J. D., Clayman, R. V. and Picus, D. D.: Comparison of endoscopic and radiological residual fragment rate following percutaneous nephrolithotripsy. J. Urol., **145**: 703, 1991.
54. Lehtoranta, K., Mankinen, P., Taari, K., Rannikko, S., Lehtonen, T. and Salo, J.: Residual stones after percutaneous nephrolithotomy; sensitivities of different imaging methods in renal stone detection. Ann Chir Gynaecol, **84**: 43, 1995.

55. Saxby, M. F., Sorahan, T., Slaney, P. and Coppinger, S. W.: A case-control study of percutaneous nephrolithotomy versus extracorporeal shock wave lithotripsy. Brit. J. Urol., **79**: 317, 1997.
56. Carr, L. K., D'A, Jewett, M. A., Ibanez, D., Ryan, M. and Bombardier, C.: New stone formation: a comparison of extracorporeal shock wave lithotripsy and percutaneous nephrolithotomy. J. Urol., **155**: 1565, 1996.
57. Lam, H. S., Lingeman, J. E., Barron, M., Newman, D. M., Mosbaugh, P. G., Steele, R. E., Knapp, P. M., Scott, J. W., Nyhuis, A. and Woods, J. R.: Staghorn calculi: analysis of treatment results between initial percutaneous nephrostolithotomy and extracorporeal shock wave lithotripsy monotherapy with reference to surface area. J. Urol., **147**: 1219, 1992.
58. Meretyk, S., Gofrit, O. N., Gafni, O., Pode, D., Shapiro, A., Verstandig, A., Sasson, T., Katz, G. and Landau, E. H.: Complete staghorn calculi: random prospective comparison between extracorporeal shock wave lithotripsy monotherapy and combined with percutaneous nephrostolithotomy. J. Urol., **157**: 780, 1997.
59. Netto, N. J., De, A. F. and Ferreira, U.: Is Percutaneous Monotherapy for Staghorn Calculus Still Indicated in the Era of Extracorporeal Shockwave Lithotripsy? J Endourol, **8**: 195, 1994.
60. Stanley, K. E. and Winfield, H. N.: Management of staghorn calculi: percutaneous nephrolithotripsy versus extracorporeal shock-wave lithotripsy. [Review] [65 refs]. Semin Urol, **12**: 15, 1994.
61. Prajsner, A., Szkodny, A., Szewczyk, W., Noga, A. and Szkodny, G.: Long-term results of kidney staghorn stone treatment with percutaneous nephrolithotripsy and extracorporeal shock-wave lithotripsy. Int Urol Nephrol, **25**: 533, 1993.
62. Saad, F., Faucher, R., Mauffette, F., Paquin, J. M., Perreault, J. P. and Valiquette, L.: Staghorn calculi treated by percutaneous nephrolithotomy: risk factors for recurrence. Urology, **41**: 141, 1993.
63. Patterson, D. E., Segura, J. W. and LeRoy, A. J.: Long-term follow-up of patients treated by percutaneous ultrasonic lithotripsy for struvite staghorn calculi. J Endourol, **1**: 177, 1987.
64. Streem, S. B. and Lammert, G.: Long-term efficacy of combination therapy for struvite staghorn calculi. J. Urol., **147**: 563, 1992.
65. Streem, S. B.: Long-term incidence and risk factors for recurrent stones following percutaneous nephrostolithotomy or percutaneous nephrostolithotomy/extracorporeal shock wave lithotripsy for infection related calculi. J. Urol., **153**: 584, 1995.
66. Cranidis, A. I., Karayannis, A. A., Delakas, D. S., Livadas, C. E. and Anezinis, P. E.: Cystine stones: the efficacy of percutaneous and shock wave lithotripsy. Urol Int, **56**: 180, 1996.
67. Bhatta, K. M., Prien, E. J. and Dretler, S. P.: Cystine Calculi- Rough and Smooth: A new clinical distinction. J. Urol., **142**: 937, 1989.
68. Knoll, L. D., Segura, J. W., Patterson, D. E., LeRoy, A. J. and Smith, L. H.: Long-Term Follow-up in Patients with Cystine Urinary Calculi Treated by Percutaneous Ultrasonic Lithotripsy. J. Urol., **140**: 246, 1988.
69. Bowsher, W. G., Blott, P. and Whitfield, H. N.: Radiation protection in percutaneous renal surgery. Brit. J. Urol., **69**: 231, 1992.
70. Inglis, J. A., Tolley, D. A. and Law, J.: Radiation safety during percutaneous nephrolithotomy. Brit. J. Urol., **63**: 591, 1989.
71. Eshghi, M., Schiff, R. G. and Smith, A. D.: Renal effects of percutaneous stone removal. Urology, **33**: 120, 1989.
72. Ishibashi, M., Morita, S., Rabito, C. A., Umezaki, N., Matsuoka, K., Noda, S., Eto, K. and Ohtake, H.: Evaluation of the therapeutic effect of percutaneous nephroureterolithotomy by Tc-99m diethylenetiaminepentaacetic acid (DTPA) renal scintigraphy--alteration of the renal fraction of blood flow, split-GFR, and renal mean transit time. Kurume Med J, **37**: 285, 1990.
73. Balbay, M. D., Varoglu, E., Devrim, H., Sahin, A., Atan, A., Ergen, A. and Remzi, D.: Quantitative evaluation of renal parenchymal mass with 99m technetium dimercapto-succinic acid scintigraphy after nephrolithotomy. J. Urol., **157**: 1226, 1997.
74. Sakkas, G., Becopoulos, T., Karayannis, A., Drossos, G. and Giannopoulou, K.: Enzymatic evaluation of renal damage caused by different therapeutic procedures for kidney stone disease. Int Urol Nephrol, **27**: 669, 1995.
75. Lechevallier, E., Siles, S., Ortega, J. C. and Coulange, C.: Comparison by SPECT of renal scars after extracorporeal shock wave lithotripsy and percutaneous nephrolithotomy. J Endourol, **7**: 465, 1993.
76. Wilson, W. T., Husmann, D. A., Morris, J. S., Miller, G. L., Alexander, M. and Preminger, G. M.: A comparison of the bioeffects of four different modes of stone therapy on renal function and morphology. J. Urol., **150**: 1267, 1993.
77. Stoller, M. L., Wolf, J. J. and St, L. A.: Estimated blood loss and transfusion rates associated with percutaneous nephrolithotomy [see comments]. J. Urol., **152**: 1977, 1994.
78. Segura, J. W., Patterson, D. E., LeRoy, A. J., Williams, H. J., Barrett, D. M., Benson, R. J., May, G. R. and Bender, C. E.: Percutaneous removal of kidney stones: review of 1,000 cases. J. Urol., **134**: 1077, 1985.
79. Patterson, D. E., Segura, J. W., LeRoy, A. J., Benson, R. J. and May, G.: The etiology and treatment of delayed bleeding following percutaneous lithotripsy. J. Urol., **133**: 447, 1985.
80. Laor, E., Palmer, L. S., Maldonado, J. R., Tolia, B. M., Karwa, G. and Reid, R. E.: Haemostatic Malecot tamponade catheter for bleeding nephrolithotomy tracts. Brit. J. Urol., **74**: 247, 1994.
81. Henriksson, C., Geterud, K., Pettersson, S. and Zachrisson, B. F.: Use of a tamponade catheter in the bleeding nephrostolithotomy track. Scand J Urol Nephrol Suppl, **138**: 15, 1991.

82. Carson, C. C. and Nesbitt, J. A.: Peritoneal extravasation during percutaneous lithotripsy. J. Urol., **134**: 725, 1985.
83. Dimberg, M., Norlen, H., Hoglund, N. and Allgen, L. G.: Absorption of irrigating fluid during percutaneous transrenal lithotripsy. Scand J Urol Nephrol, **27**: 463, 1993.
84. Lloyd, S. N., Kirk, D., Deane, R. F. and Kyle, K. F.: Effect of percutaneous nephrolithotomy on thermoregulation. Brit. J. Urol., **69**: 132, 1992.
85. O'Donnell, A., Schoenberger, C., Weiner, J. and Tsou, E.: Pulmonary complications of percutaneous nephrostomy and kidney stone extraction. South Med J, **81**: 1002, 1988.
86. LeRoy, A. J., Williams, H. J., Bender, C. E., Segura, J. W., Patterson, D. E. and Benson, R. C.: Colon perforation following percutaneous nephrostomy and renal calculus removal. Radiology, **155**: 83, 1985.
87. Vallancien, G., Capdeville, R., Veillon, B., Charton, M. and Brisset, J. M.: Colonic perforation during percutaneous nephrolithotomy. J. Urol., **134**: 1185, 1985.
88. Ahmed, M. and Reeve, R.: Iatrogenic duodeno-cutaneous fistula at percutaneous nephrolithotomy managed conservatively. Brit. J. Urol., **75**: 416, 1995.
89. Kondas, J., Szentgyorgyi, E., Vaczi, L. and Kiss, A.: Splenic injury: a rare complication of percutaneous nephrolithotomy. Int Urol Nephrol, **26**: 399, 1994.
90. Green, D. F., Lytton, B. and Glickman, M.: Ureteropelvic junction obstruction after percutaneous nephrolithotripsy. J. Urol., **138**: 599, 1987.
91. Chang, C. P., Chen, M. T. and Chang, L. S.: Autonomic hyperreflexia in spinal cord injury patient during percutaneous nephrolithotomy for renal stone: a case report. J. Urol., **146**: 1601, 1991.
92. Ahn, J., Trost, D. W., Topham, S. L. and Sos, T. A.: Retained nephrostomy thread providing a nidus for atypical renal calcification. Br J Radiol, **70**: 309, 1997.
93. Marberger, M., Stackl, W., Hruby, W. and Kroiss, A.: Late sequelae of ultrasonic lithotripsy of renal calculi. J. Urol., **133**: 170, 1985.
94. Ivory, C. M., Dubbins, P. A., Wells, I. P. and Hammonds, J. C.: Ultrasound assessment of local complications of percutaneous renal stone removal. J Clin Ultrasound, **17**: 345, 1989.
95. Page, J. E. and Walker, W. J.: Complications attributable to the formation of the track in patients undergoing percutaneous nephrolithotomy. Clin Radiol, **45**: 20, 1992.
96. Moskowitz, G. W., Lee, W. J. and Pochaczevsky, R.: Diagnosis and management of complications of percutaneous nephrolithotomy. Crit Rev Diagn Imaging, **29**: 1, 1989.
97. Lee, W. J., Smith, A. D., Cubelli, V., Badlani, G. H., Lewin, B., Vernace, F. and Cantos, E.: Complications of percutaneous nephrolithotomy. AJR Am J Roentgenol, **148**: 177, 1987.
98. Timmons, J. W., Malek, R. S., Hattery, R. R. and Deweerd, J. H.: Caliceal diverticulum. J. Urol., **114**: 6, 1975.
99. Cohen, T. D. and Preminger, G. M.: Management of calyceal calculi. [Review] [85 refs]. Urol Clin North Am, **24**: 81, 1997.
100. Psihramis, K. E. and Dretler, S. P.: Extracorporeal Shock Wave Lithotripsy of Caliceal Diverticula Calculi. J. Urol., **138**: 707, 1987.
101. Bellman, G. C., Silverstein, J. I., Blickensderfer, S. and Smith, A. D.: Technique and follow-up of percutaneous management of caliceal diverticula. Urology, **42**: 21, 1993.
102. Hendrikx, A. J., Bierkens, A. F., Bos, R., Oosterhof, G. O. and Debruyne, F. M.: Treatment of stones in caliceal diverticula: extracorporeal shock wave lithotripsy versus percutaneous nephrolitholapaxy. Brit. J. Urol., **70**: 478, 1992.
103. Grasso, M., Lang, G., Loisides, P., Bagley, D. and Taylor, F.: Endoscopic management of the symptomatic caliceal diverticular calculus. J. Urol., **153**: 1878, 1995.
104. Wolf, J. J. and Clayman, R. V.: Percutaneous nephrostolithotomy. What is its role in 1997?. [Review] [64 refs]. Urol Clin North Am, **24**: 43, 1997.
105. Minon, C. J., Garcia, T. E., Garcia, D.E., Vela, N. R., Alda, A., Plaza, J. and Alferez, C.: Percutaneous nephrolithotomy in transplanted kidney. Urology, **38**: 232, 1991.
106. Fahlenkamp, D., Oesterwitz, H., Althaus, P., Schopke, W. D. and Brien, G.: Percutaneous management of urolithiasis after kidney transplantation. Report of a case and review of the literature. [Review] [19 refs]. Eur Urol, **14**: 330, 1988.
107. Gedroyc, W. M., MacIver, D., Joyce, M. R. and Saxton, H. M.: Percutaneous stone and stent removal from renal transplants. Clin Radiol, **40**: 174, 1989.
108. Bailey, I. S., Griffin, P., Evans, C. and Matthews, P. N.: Percutaneous surgery of the transplanted kidney. Brit. J. Urol., **63**: 327, 1989.
109. Jones, D. J., Kellett, M. J. and Wickham, J. E.: Percutaneous nephrolithotomy and the solitary kidney. J. Urol., **145**: 477, 1991.
110. Jones, D. J., Wickham, J. E. and Kellett, M. J.: Percutaneous nephrolithotomy for calculi in horseshoe kidneys. J. Urol., **145**: 481, 1991.
111. Cussenot, O., Desgrandchamps, F., Ollier, P., Teillac, P. and Le, D. A.: Anatomical bases of percutaneous surgery for calculi in horseshoe kidney. Surg Radiol Anat, **14**: 209, 1992.
112. Toth, C., Holman, E., Pasztor, I. and Khan, A. M.: Laparoscopically controlled and assisted percutaneous transperitoneal nephrolithotomy in a pelvic dystopic kidney. J Endourol, **7**: 303, 1993.
113. Watson, D. L., Green, L. S. and Loughlin, K. R.: 'Open' percutaneous ultrasonic lithotripsy for failed anatrophic nephrolithotomy. Brit. J. Urol., **75**: 249, 1995.

114. Carson, C. C., Danneberger, J. E. and Weinerth, J. L.: Percutaneous lithotripsy in morbid obesity. J. Urol., **139**: 243, 1988.
115. Giblin, J. G., Lossef, S. and Pahira, J. J.: A modification of standard percutaneous nephrolithotripsy technique for the morbidly obese patient. Urology, **46**: 491, 1995.
116. Stoller, M. L., Bolton, D., St, L. M. and Lawrence, M.: Percutaneous nephrolithotomy in the elderly. Urology, **44**: 651, 1994.
117. Horgan, J. D., Maidenberg, M. J. and Smith, A. D.: Endopyelotomy in the elderly [see comments]. J. Urol., **150**: 1107, 1993.
118. Kurzrock, E. A., Huffman, J. L., Hardy, B. E. and Fugelso, P.: Endoscopic treatment of pediatric urolithiasis. J Pediatr Surg, **31**: 1413, 1996.
119. Burns, J. R. and Joseph, D. B.: Combination therapy for a partial staghorn calculus in an infant. J Endourol, **7**: 469, 1993.
120. Surya, B. V., Rothberg, M., Schwarzman, M. and Johanson, K. E.: Management of simple renal cysts during percutaneous nephrostolithotomy. Urology, **33**: 472, 1989.
121. Kropp, B. P., Dabagia, M. D., Scott, J. W. and Lingeman, J. E.: Percutaneous nephrolithotomy directly through an angiomyolipoma. Urology, **44**: 915, 1994.
122. Chandhoke, P. S.: Cost-effectiveness of different treatment options for staghorn calculi [comment]. J. Urol., **156**: 1567, 1996.

**This article should be referenced as follows:**

Ramakumar, S. and Segura, J.W. (2004) Percutaneous management of urinary calculi. *TheScientificWorldJOURNAL* **4 (S1)**, 296–307.

**Handling Editor:**

Anthony Atala, Principle Editor for *Urology* — a domain of *TheScientificWorldJOURNAL*.
